# A 24 month follow-up of refractory macular holes treated with an autologous transplantation of internal limiting membrane versus retina expansion technique

**DOI:** 10.1186/s40942-021-00329-1

**Published:** 2021-10-02

**Authors:** Arturo Alezzandrini, Camila I. Dorrego, María Victoria Cibrán, Valentina Cortina-Revelli, Franco D. Rocco, Marcelo Zas, Lihteh Wu

**Affiliations:** 1 Oftalmos Centro Oftalmológico de Alta Complejidad, University of Buenos Aires, Buenos Aires, Argentina; 2 Sanatorio Otamendi y Miroli, University of Buenos Aires, Buenos Aires, Argentina; 3 Hospital de Clínicas José de San Martin, University of Buenos Aires, Buenos Aires, Argentina; 4 Asociados de Macula, Vitreo y Retina de Costa Rica, San Jose, Costa Rica

**Keywords:** Macular holes, Hydrodissection, ILM free flap, Retina expansion

## Abstract

**Background:**

To compare the functional and anatomic outcomes at 24 months of eyes with a primary macular hole that failed to close after a prior surgery and were treated with either an autologous transplantation of internal limiting membrane (AT-ILM) or the retina expansion (RE) technique.

**Methods:**

Retrospective, single center, comparative study of 28 eyes with a macular hole that failed to close after a prior vitrectomy. All eyes had a size of ≥ 500 μm. Participants were divided into two groups according to the type of intervention performed: AT-ILM group (n = 14) and RE group (n = 14). Main outcomes measured were the MH closure rate assessed by spectral-domain optical coherence tomography (SD-OCT) and the best-corrected visual acuity (BCVA) at 24 months after surgery.

**Results:**

Patients in the AT-ILM group experienced a statistically significantly improved post-operative BCVA (median 49.50 letters, range 20–66 letters) over the pre-operative BCVA (median 39 letters, range 18–52 letters) (p-value = 0.006 Wilcoxon paired sample test). In contrast, patients in the RE group did not achieve a statistically significant improvement (p-value = 0.328, Wilcoxon paired sample test). The median pre-operative BCVA was 35 letters (range 18–52 letters), whereas the median post-operative BCVA was 39 letters (range 16–66 letters). At 24 months of follow-up, 85.7% of patients in the AT-ILM group achieved closure compared to 57.1% in the RE group (p-value = 0.209, Fisher’s exact test). Multivariate analysis showed that MH size and baseline BCVA were important determinants of post-operative BCVA. The baseline MH size was the only significant pre-operative factor that influenced MH closure.

**Conclusions:**

This study demonstrates similar closure rates for both groups however better visual outcomes were obtained with the AT-ILM.

**Supplementary Information:**

The online version contains supplementary material available at 10.1186/s40942-021-00329-1.

## Introduction

In 1991 Kelly and Wendel [1] pioneered vitrectomy surgery for macular holes (MH), a previously untreatable condition. They showed that a vitrectomy with the extraction of the posterior hyaloid followed by gas tamponade with a non-expansile concentration obtained an anatomic success rate of 58%. Since then, this technique has evolved over the years. Currently most vitreoretinal surgeons combine pars plana vitrectomy (PPV) with peeling of the internal limiting membrane (ILM) [2]. Current anatomic success rates for the repair of primary idiopathic MH ≤ 400 μm in size range between 90 and 98%. In contrast idiopathic MH ≥ 400 μm remain a challenge for all vitreoretinal surgeons [3]. MH size is based on the horizontal linear width measured at the narrowest point of the hole [4].

Several pre-operative factors that influence the final post-operative outcome have been described. These include the diameter of the MH, the pre-operative visual acuity, the number of prior surgeries, the hole conformation and the choroidal thickness [5]. The morphological configuration of the sealed MH has also been shown to have a prognostic implication in the post-operative visual acuity. Better results have been found in those holes that recovered the normal foveal contour without exposure of the underlying retinal pigment epithelium. Those holes with a “U” shape, which are present in approximately 45% of cases, are associated with a better functional and anatomical outcome. In contrast, MH with flat edges, whose incidence is between 19 and 39%, are associated with poorer functional results [6].

In 2010 Michalewska and collaborators [7] compared the inverted ILM flap technique to the conventional ILM peel technique in eyes with MH ≥ 400 μm. The eyes that underwent the inverted ILM flap technique obtained better anatomical and functional outcomes than those that were operated with the conventional technique [7, 8]. Unfortunately this technique cannot be applied in cases where the ILM has been removed during previous surgery. To address this issue, different surgical techniques have been developed including the retinal expansion (RE) and the autologous transplantation of the ILM (AT-ILM) technique [2, 9, 10]. In the RE technique, balanced saline solution is injected subretinally around the MH until a macular detachment surrounding the MH is formed [9, 10]. In the AT-ILM technique, an ILM-free flap is harvested and placed inside the macular hole where it serves as a scaffold, stimulating cell proliferation and restoring the foveal structure [2]. The purpose of the current pilot study was to compare the anatomic and functional outcomes of the AT-ILM with the RE technique in eyes with a primary MH that did not close after a prior surgery.

## Methods

This retrospective, comparative pilot study included 28 patients with a primary MH that did not close after a primary surgery. These patients underwent a second vitrectomy with either the AT-ILM or the RE techniques. All patients were seen at the vitreoretinal service of the Instituto de Alta Complejidad Oftalmos, Buenos Aires, Argentina, from December 2016 to December 2017. The study was approved by the local IRB. Written informed consent was obtained from all the patients. This study adhered to the tenets of the Declaration of Helsinki.

### Patient eligibility and exclusion criteria

All patients with a MH that underwent surgical repair were included in the study if they met the following criteria: (1) prior surgical failure to repair a primary MH; (2) MH with a size of ≥ 500 μm; (3) No other possible causes for visual loss.

The size and basal diameter of the MH were measured using the caliper function in the Spectralis OCT (Heidelberg Engineering, Heidelberg, Germany) software. The basal hole diameter of the MH was measured as the linear length of foveal detachment. MH size was based on the horizontal linear width measured at the narrowest point of the hole as described by the International Vitreomacular Traction Study Group [4].

Patients were excluded if they had a history of diabetic retinopathy, or if they exhibited high myopia (spherical equivalence ≥ 6 D or axial length ≥ 26.5 mm) or retinal detachment associated with MH or epiretinal membranes.

### Examination and treatment procedures

At baseline and at each scheduled post-operative visit, each patient underwent a complete ophthalmic examination and spectral domain optical coherence tomography (SD-OCT). A volume scan centered on the fovea was performed. The scans were reviewed and manual corrections were performed in case of segmentation errors.

### Surgical procedures

All eyes underwent prior PPV for repair of a primary MH. The second procedure was performed by three differents surgeons (AA, CM and MZ) using the 23-gauge vitrectomy Constellation (Alcon Laboratories, Fort Worth, Texas, USA) and the NGENUITY® 3D visualization system (Alcon Laboratories). If significant lens opacification was present, phacoemulsification and intraocular lens implantation was jointly performed (10 eyes).

Intravitreal triamcinolone was used to verify that the posterior hyaloid had been peeled. ILM-Blue® 0.025% (DORC, Zuidland, the Netherlands) was injected over the macular area to verify that there was no residual ILM at the edges of the MH. In eyes that underwent autologous transplantation of the ILM (Additional file 1: Video S1 available), a new ILM rhexis of 2 disc areas was performed under perfluoro-octane (Perfluoron, Alcon Laboratories) with a 23 G Finesse Flex Loop (Alcon Laboratories). The ILM removal was completed with the Constellation pneumatic DSP handpiece (Alcon Laboratories) and 23G ILM forceps tip (Alcon Laboratories). The new ILM free flap was gently laid over the MH. A perfluoro-octane-air exchange was completed. This was followed by an air-14% perfluoropropane C3F8 (ISPAN, Alcon Laboratories) exchange. Face-down posturing was advised for about one week after the surgery.

In eyes that underwent the RE technique (Additional file 1: Video S1 available), the macula was stained with 0.025% ILM-Blue® (DORC) to verify that there was no residual ILM present. A small bubble of perfluoro-octane (Alcon Laboratories) was injected over the MH and them BSS Balanced Salt Solution (Alcon Laboratories) was injected into the subretinal perimacular space using a 23 g/38 g 2 mm PolyTip cannula (MedOne, Sarasota, Fl, USA) which was coupled to the automatic viscous fluid injector operated by the surgeon with the foot pedal. Two injections were made around the macular hole (one superior to the macular hole and one inferior to the macular hole). The injection of BSS subretinally was performed to promote retinal detachment and retinal stretching of the area around the MH. A partial fluid–air exchange was performed using a flute needle to removed the perfluoro-octane and to promote enlargement of the macular detachment and encouraging further stretching of the retina. BSS was re-introduced into the vitreous cavity to enable manual massage of the retina. Finally, a complete air-14% perfluoropropane (C3F8) (ISPAN, Alcon Laboratories) exchange was completed. Face-down posturing was advised for about one week after the surgery.

### Data analysis

The main outcomes measured were MH closure and the post-operative best corrected visual acuity (BCVA). MH closure was defined as flattening of the retinal detachment around the MH regardless of the configuration. Snellen visual acuities were transformed to the ETDRS letter score to facilitate the statistical analysis.

Statistical analysis was performed using R (version 3.6.2, The R Foundation for Statistical Computing, available at https://cran.r-project.org). For quantitative variables, the mean and standard deviation were calculated after evaluating their normality using the Shapiro-Wilk test. For categorical variables, absolute frequencies and percentages were calculated. To compare median visual acuity before and after surgery, the Wilcoxon test for paired samples was used. To evaluate the possible association between the status of the MH and the type of surgery, a Fisher’s exact test was performed. A p-value ≤ 0.05 was considered statistically significant. Univariate and multivariate linear regression models with backward elimination were built to identify variables that influenced post-operative visual acuity and MH closure. For the variables retained in the final model, the corresponding odds ratios and their 95% confidence intervals (CI 95%) were calculated.

## Results

### Baseline characteristics

A total of 28 eyes from 28 patients fulfilled the inclusion and exclusion criteria.

Table 1 compares the baseline characteristics between the AT-ILM and RE groups. There were no statistically significant differences between the groups (Table 1).

**Table 1:** Baseline characteristics of patients

Variable	Treatment		p-value
	AT-ILM (n = 14)	RE (n = 14)	
Gender = male (%)	7 (50.00)	5 (35.70)	0.703
Hole size (µm)	640.79 ± 94.75	646.43 ± 99.15	0.879
BCVA (ETDRS letters)	35.9 ± 11.7	38.2 ± 9.5	0.563
Time between first and second surgeries (months)	11.3 ± 3.2	10.8 ± 2.5	0.5376

### Visual acuity outcomes

**Fig. 1:**
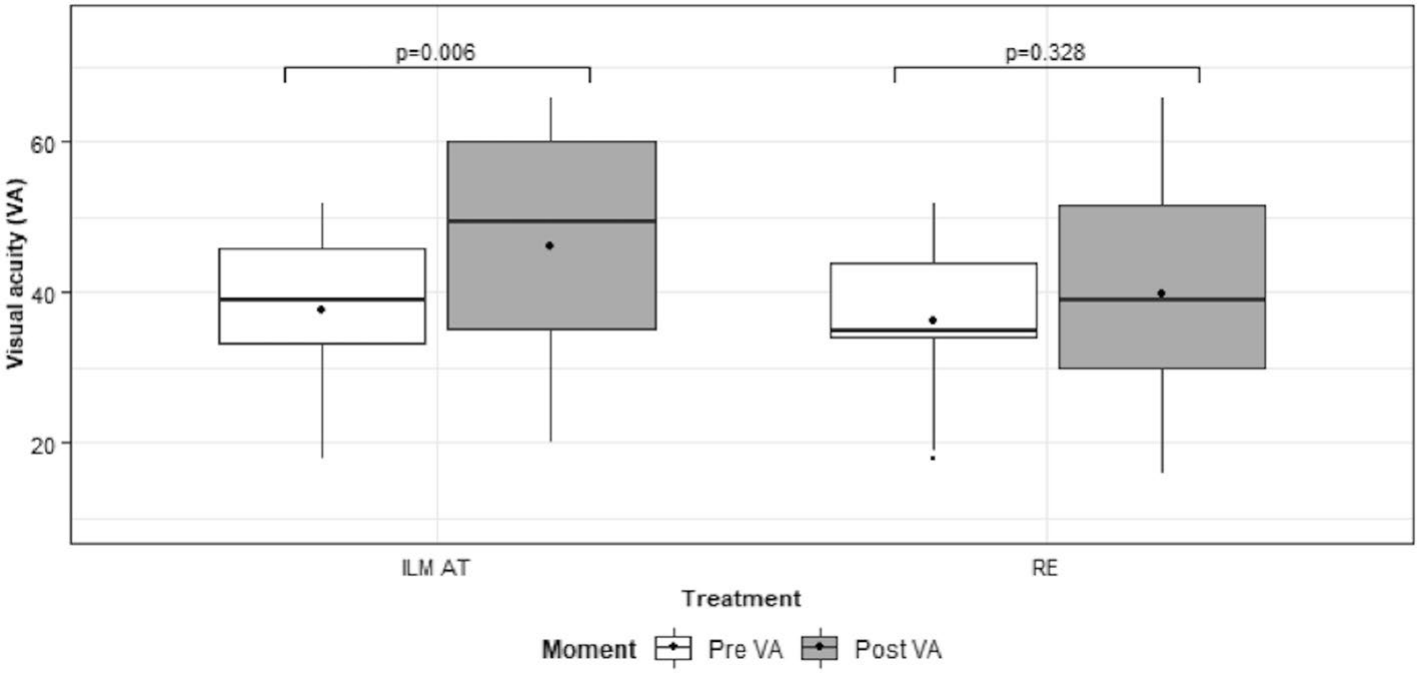
Pre-operative and post-operative visual acuity distribution

Figure 1 shows the comparison of the pre-operative BCVA and the post-operative BCVA at 24 months by treatment group. For patients in the AT-ILM group, the post-operative BCVA (median 49.50 letters Snellen 20/100, range 20–66 letters Snellen 20/50–20/400) was statistically significantly improved (p-value = 0.006 Wilcoxon paired sample test) over the pre-operative BCVA (median 39 letters Snellen 20/160, range 18–52 letters Snellen 20/100–20/450). In contrast, patients in the RE group did not achieve a statistically significant improvement (p-value = 0.328, Wilcoxon paired sample test). The median pre-operative BCVA was 35 letters Snellen 20/200 (range 18–52 letters Snellen 20/90–20/450), whereas the median post-operative BCVA was 39 letters Snellen 20/160 (range 16–66 letters Snellen 20/50–20/500).

Table 2 summarizes the results of the adjustment of linear regression models where the dependent variable is post-operative VA. Univariate analysis identified MH size (p < 0.001), the time between surgeries (p = 0.001) and baseline pre-operative BCVA (p < 0.001) as statistically significant variables that influenced post-operative BCVA. Multivariate analysis showed that MH size and baseline BCVA were statistically significant (p-value < 0.001 in both cases). According to the estimated model, for each micron of increase in hole size, the mean post-operative BCVA decreased by -0.087 letters (CI 95% − 0.118; − 0.056), and for each unit of increase in baseline BCVA, the mean post-operative BCVA increased by 0.779 letters (CI 95% 0.505; 1.053).

**Table 2:** Linear regression model with post-operative VA as the dependent variable

Variable	Univariate Model		Multivariate Backward Model	
	Estimate (SE)	p-value	Estimate (SE)	p-value
Sex (male vs. female)	9.188 (5.917)	0.133		
Macular hole size	− 0.139 (0.018)	< 0.001	− 0.087 (0.016)	< 0.001
Baseline BCVA	1.262 (0.163)	< 0.001	0.779 (0.140)	< 0.001
Time between first and second vitrectomy	− 3.217 (0.901)	0.001		

*BCVA *best corrected visual acuity, *SE *standard error

### Anatomic results

**Fig. 2:**
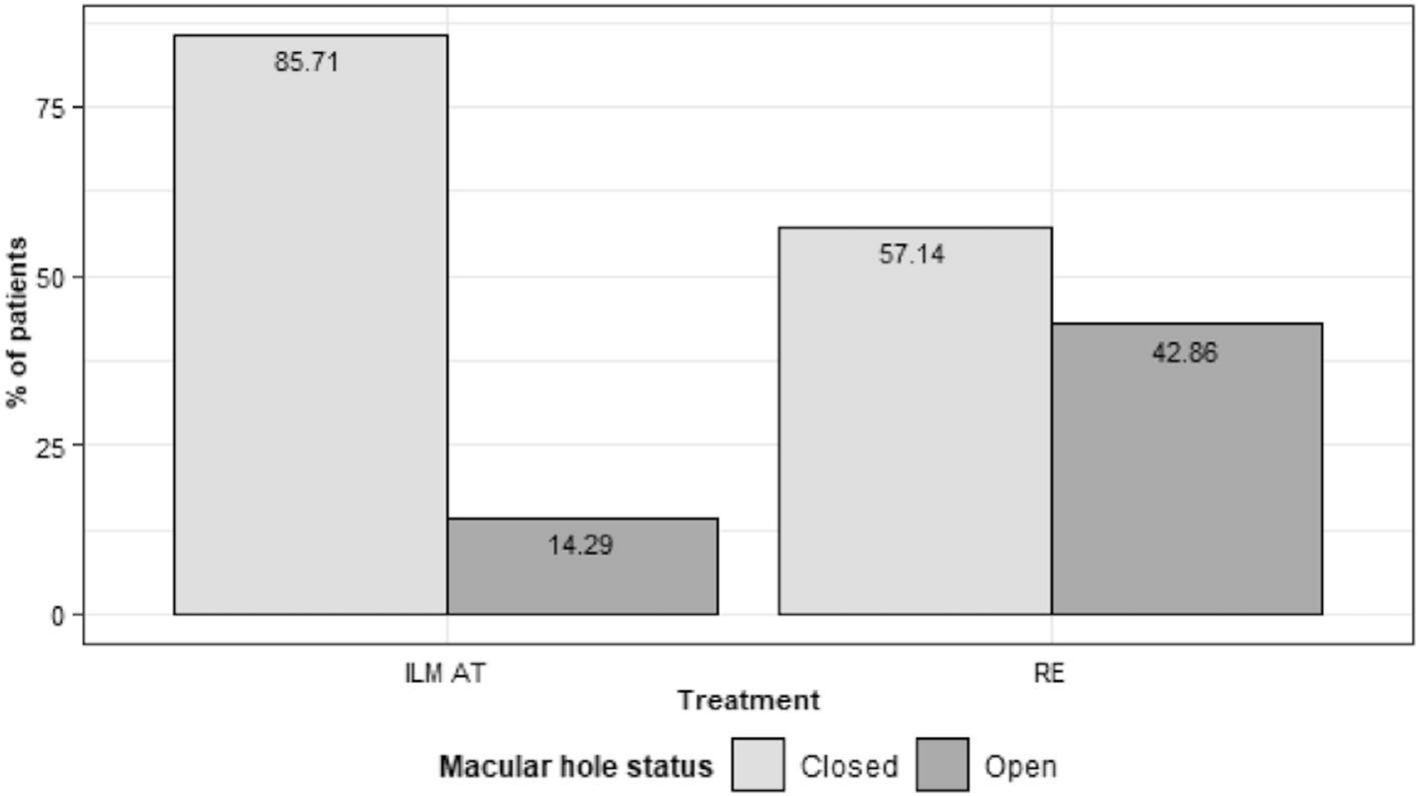
Post-operative anatomic macular hole status

At 24 months of follow-up, 85.7% of patients in the AT-ILM group achieved closure compared to 57.1% in the RE group (Fig. 2, p-value = 0.209 Fishers exact test). The closure types are summarized in Table 3. In the AT-ILM group more eyes achieved a U and V shaped closure compared to the RE group.

**Table 3:** Type of anatomic closure based on SD-OCT

Type of closure	AT-ILM	RE
U Shaped	5	2
V Shaped	3	1
Irregular	1	5
Open	2	6

*SD-OCT* spectral domain optical coherence tomography

Table 4 summarizes the results of the adjusted logistic regression models to analyse the relationship between the anatomic status of the macular hole and the other variables. Univariate analysis identified MH size (p = 0.009) and time elapsed between surgeries (p = 0.015). Multivariate analysis showed that only the MH size (p = 0.009) remained statistically significant for MH closure. According to the estimated model, the odds of having a closed macular hole decreased 2% (OR: 1.02, CI 95% 1.01–1.05) for each µm increase in the baseline MH hole size.

**Table 4:** Linear regression model with anatomic status of MH as the dependent variable

Variable	Univariate model		Multivariate backward model	
	Estimate (SE)	p-value	Estimate (SE)	p-value
Gender	− 0.511 (0.966)	0.597	NA	NA
Macular hole size	0.023 (0.009)	0.009	0.023 (0.009)	0.009
Baseline Visual Acuity	− 0.065 (0.043)	0.136	NA	NA
Time between first and second vitrectomy	0.704 (0.290)	0.015	NA	NA

*MH*  macular hole, *SE* standard error, *NA* not applicable

## Discussion

Modern MH repair with PPV, posterior hyaloid detachment and ILM peeling with gas tamponade and post-operative face-down positioning results in closure of most of the MH ≤ 400 μm in size. Larger and chronic MH have a much lower closure rate. Several techniques, including RE and AT-ILM among others, have been developed to address larger holes and those that have failed primary surgical repair [2, 11].

The RE technique was originally described by Oliver and Wojcik [9] in 2011. The objective behind this technique is to separate the retina surrounding the MH from the underlying RPE. The rationale of this technique is that once the retina is detached, it becomes more compliant and its edges are easier to approximate facilitating closure of the MH. Wong et al. [12] used the RE technique to operate on 16 patients with a MH ≥ 650 μm. They reported that 83% of eyes achieved MH closure. However the mean gain of BCVA was only of 0.04 logMAR. In contrast, Felfeli and Mandelcorn [13] reported that they were able to close the MH in 87% of their 39 eyes with 80% of those eyes gaining ≥ 2 lines of BCVA. In Meyer and co-workers’ [14] case series of 41 eyes, closure was achieved in 85% of eyes. The mean post-op BCVA improved from 20/200 at baseline to 20/91 at the last follow-up. In 9 of the 10 eyes of Szigiato et al’s [15] series, the MH achieved closure and the mean improvement of BCVA was 16 letters. The average MH diameter was 654 μm, however it remains unclear what they mean by the diameter.

Morizane et al. [16] developed the AT-ILM technique with the objective of placing a scaffold over the MH to facilitate MH closure. In their pilot study of 10 eyes that included large chronic MH, traumatic MH, myopic foveoschisis, optic pit foveoschisis and proliferative diabetic retinopathy, they were able to close 90% of the MH. The visual outcomes were also favorable with 8 eyes improving more than 0.2 logMAR units [16]. De Novelli and colleagues [17] obtained similar results in another small series of 10 eyes. Yuan et al. [18] reported that quality of life measures also improved following MH closure using the AT-ILM technique.

Our anatomic results are in line with all these prior studies. We observed a similar anatomical outcome between both techniques. In contrast the functional outcome was better in eyes that underwent ILM-AT when compared to the RE technique. The type of anatomic closure has been associated with the post-operative visual acuity [19, 20]. In our study more eyes in the AT-ILM group achieved a U or V shaped closure than the eyes in the RE group. Conversely more eyes in the RE group had an irregular shaped closure. Alternatively, the fluid wave used to create the retinal detachment and retinal stretching around the macular hole may have caused some mechanical trauma to the photoreceptors. The numbers were too small to perform statistical analysis. Frisina and colleagues [11] recently reviewed 10 different surgical techniques used to manage these MH. They concluded that closure rates were similar in all of these techniques. However the visual gains differed depending on the surgical technique used to repair the refractory MH. In their review they concluded that the use of human amniotic membrane gave the best visual acuity results. They argued that RE required a complex and unjustified surgical maneuver [11].

Limitations of the current study include its retrospective design and small number of cases. Furthermore BCVA may not be the best functional parameter to assess in these eyes with poor BCVA. Therefore future studies should also include microperimetric data and quality of life measures to determine the efficacy and value of both techniques.

## Conclusions

In summary, both RE and AT-ILM allow closure of refractory MH. The closure rates are similar between both techniques but the type of closure may differ between techniques which may explain in part the better visual acuity outcomes obtained with AT-ILM. Multivariate analysis showed that MH size was the only determinant of post-operative BCVA. The time elapsed between surgeries was the only significant pre-operative factor that influenced MH closure.

## Supplementary Information

**Additional file 1.** Videos illustrating the autologous transplantation of internal limiting membrane and the retina expansion techniques for refractory macular holes.

## Data Availability

The datasets used and/or analysed during the current study are available from the corresponding author on reasonable request.
